# The Impact of Fear of Missing out (FoMO) on Addictive Eating: A Moderated Mediation Model

**DOI:** 10.3390/nu18101493

**Published:** 2026-05-08

**Authors:** Janelle A. Skinner, Rebecca A. Collins, Kerith Duncanson, Phillipa J. Hay, Tracy L. Burrows

**Affiliations:** 1 School of Health Sciences, College of Health, Medicine and Wellbeing, University of Newcastle, Callaghan, NSW 2308, Australia; rebecca.collins10@newcastle.edu.au (R.A.C.); tracy.burrows@newcastle.edu.au (T.L.B.); 2 Hunter Medical Research Institute, New Lambton Heights, NSW 2305, Australia; kerith.duncanson@newcastle.edu.au; 3 School of Medicine and Public Health, College of Health, Medicine and Wellbeing, University of Newcastle, Callaghan, NSW 2308, Australia; 4 Translational Health Research Institute, Western Sydney University, Campbelltown, NSW 2751, Australia; p.hay@westernsydney.edu.au; 5 Mental Health Services, Camden and Campbelltown Hospitals, South Western Sydney Local Health District (SWSLHD), Campbelltown, NSW 2751, Australia

**Keywords:** fear of missing out, addictive eating, reward-related eating, mental health, anxiety, mediation analyses

## Abstract

Background/Objectives: Fear of missing out (FoMO) describes the concern of missing out on a rewarding experience, a contemporary psychological phenomenon that has yet to be explored in relation to addictive eating. This exploratory study examined the relationship between FoMO and addictive eating, and the effect of reward-related eating and mental health status (anxiety, depression, and stress) on the relationship. Methods: A sample of 227 adults (mean age 49.4 years; 79.3% women) completed an online survey to assess addictive eating (Yale Food Addiction Scale), FoMO (Fear of Missing Out Scale), reward-related eating (Reward-Based Eating Drive Scale), anxiety, depression, and perceived stress (GAD-7, PHQ-8, PSS-4). Mediation and moderated mediation analyses were performed to determine relationships between variables. Results: A direct relationship between FoMO and addictive eating was found. The relationship was partially mediated by reward-related eating after accounting for BMI and gender. Anxiety severity was a significant moderator of the relationship between reward-related eating levels and addictive eating symptoms. Conclusions: This study provides insights that can assist in informing interventions to mitigate the negative effects of FoMO on eating behaviours, particularly among populations vulnerable to anxiety.

## 1. Introduction

Addictive eating, originally broadly termed ‘food addiction’ and more recently ‘hyperpalatable’ or ‘ultra-processed food addiction’ [1], is theorized as being on the severe end of a spectrum of overeating [2]. It is a phenotype of eating behaviour marked by the chronic excessive and dysregulated consumption of hyperpalatable or ultra-processed energy-dense foods [1,3]. This is characterised by diminished control over consumption, craving for certain foods, and continued overeating despite negative consequences [3,4]. Past research demonstrates that the excessive intake of hyperpalatable or ultra-processed foods not only impacts an individual’s physical health, but also their mental wellbeing [5]. For example, addictive eating is positively correlated with anxiety, depression, and stress prevalence [6,7]. Further, individuals with addictive eating report overeating in response to emotional triggers like anxiety, depression, and stress [8]. Higher prevalence of addictive eating has been consistently reported in females, women, and girls compared to males, men, and boys [9,10,11]. The female sex has also been found to be a predictor of severe food addiction [12], and high reward sensitivity significantly associated with greater addictive eating symptoms in females [13]. While many factors may influence addictive eating, findings from recent qualitative research [14] in adults with addictive eating identified that the ‘fear of missing out’ (FoMO) may play a role. This study aimed to examine the contribution of FoMO to addictive eating.

### 1.1. FoMO and Addictive Eating

FoMO, first introduced in the literature to describe a phenomenon observed with use of social networking sites, is the concern of missing out on rewarding experiences [15]. It is characterised by two processes, the perception of missing out which provokes anxiety (e.g., apprehension, worry, rumination), followed by compulsive behaviour aimed at relieving anxiety (e.g., frequent checking of social media sites and messaging services to avoid missing out on rewarding experiences) [16,17]. Published research and qualitative insights from working with those with lived experience of addictive eating suggest that a similar interpretation of FoMO can be applied to food [14]. Individuals may experience anxiety or apprehension when they believe they are missing out on a unique dining experience, taste/flavor combination of new foods (e.g., highly marketed ultra-processed foods), or unable to partake in eating a food they enjoy [14]. The excitement or stimulation foods offer may provide temporary distraction or relief from FoMO. However, the more an individual engages in overeating behaviours to cope with this negative affective state, the more reinforced and habitual these behaviours may become, leading to dependence on food, i.e., addictive eating or ‘food addiction’.

Though FoMO has drawn increasing attention in the research community in recent years, it is yet to be explored in relation to addictive eating and the potential role it could have in tailoring or creating pathways that may contribute towards personalized smart nutrition management. Existing research on FOMO, predominantly cross-sectional in design, has focused on the effect of FoMO on excessive or ‘problematic’ digital technology use and psychological wellbeing assessed via self-report surveys [18]. Findings include moderate to large positive associations between FoMO and social media and smartphone use in adolescents and adults [16]. More recent studies in university students, limited by use of self-report measures, have examined FoMO in relation to night eating syndrome [19] and alcohol use [20], both of which can include binge-like behaviour in consumption patterns not unlike addictive eating [21,22]. No significant correlation between FoMO and night eating syndrome was found, but there was a small significant positive correlation between FoMO and the number of meals eaten in a day [19]. Individuals with high levels of FoMO were found to consume more alcohol on drinking occasions and were more likely to experience negative alcohol-related consequences (e.g., difficulties limiting drinks, neglecting obligations, interpersonal problems [23]) compared to those with low levels of FoMO [20]. Studies examining gender differences have found video gaming and gambling behaviours [24] linked to higher FoMO scores in men, while women are more prone to experiencing FoMO related to online social engagement, interpersonal relationships, and social expectations [25].

The neurobiological basis of FoMO has not been well explored [16,26], but it is postulated that FoMO activates brain regions associated with reward (e.g., mesolimbic systems) [27] which may trigger the release of dopamine when a person engages in activities that alleviate the anxiety associated with FoMO [17], such as checking social media or participating in social events [16,28]. In the context of addictive eating, the consumption of desired foods may have a similar reinforcing effect. Preclinical and clinical research highlights the contributions of reward systems, particularly in the dopaminergic pathway, in response to hyperpalatable or ultra-processed food intake [29,30,31]. Further, given studies have found that FoMO is related to maladaptive behaviours, FoMO may be an important, and understudied, factor to consider when it comes to addictive eating. It is plausible that a heightened intensity of FoMO may be a contributor to addictive eating.

### 1.2. Reward-Related Eating as a Mediator

Reward-related eating, defined as eating driven by the rewarding and relieving aspects of food such as the desire for emotional gratification or to alleviate negative emotions (e.g., stress, boredom) [32,33], echoes the characteristics of addictive eating behaviours although is thought to be on the milder end of the spectrum [34]. Reward-related eating involves several subcomponents, such as preoccupation with food-related thoughts, loss of control during eating, and lack of satiety [35]. This multidimensional construct that reflects interconnected cognitive, emotional, and physiological responses to food-related cues [36], shares significant overlap with other maladaptive eating behaviours. For example, emotional eating and stress-induced eating, where individuals are more prone to experience loss of control over eating where the urge to eat becomes an automatic response to cognitions and emotions rather than in response to physical hunger cues [33,36]. It has been proposed that repeated reward-related eating, particularly in response to emotional states in which short-term pleasure is derived from eating food, can form the basis of habitual overeating that may be an antecedent to increased severity of binge eating and, at the extreme, addictive eating [31,34]. While independently it is hypothesized that FoMO will predict addictive eating, it may be that reward-related eating will mediate this relationship as individuals who experience higher levels of FoMO may be more likely to engage in rewarding behaviours, and as suggested, reward-related eating may be an antecedent to addictive eating.

### 1.3. Mental Health Status as a Moderator

While FoMO may directly or indirectly be related to addictive eating, some differences might be observed in these relationships depending on the level of anxiety or depression or perceived stress experienced by individuals. Higher levels of FoMO, dependent and independent of technology use, has been found to be associated with increased mental health symptomology and severity (e.g., anxiety, depression, and psychological stress) [25,37,38] and other indicators of poor psychological wellbeing [16,18] (e.g., lower general mood, lower life satisfaction, lower emotional wellbeing [15,39,40,41]). Moreover, anxiety and depression have been found to predict the extent an individual experiences FoMO [42]. In individuals with higher levels of anxiety, depression, or perceived stress, the negative effect caused by FoMO may impede coping mechanisms and amplify maladaptive eating behaviours. For example, the use of food as a reward or to self-soothe in response to life stressors.

### 1.4. Hypotheses

**Figure 1:**
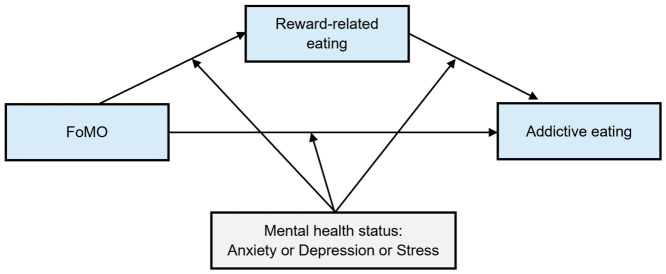
Hypothesised moderated mediation model. This figure exhibits the proposed relationship among fear of missing out (FoMO), reward-related eating and addictive eating. Reward-related eating is hypothesised to mediate the relationship between FoMO and addictive eating, while mental health status is hypothesised to moderate these relationships.

Thus, the current study proposed a moderated mediation model to explore (1) the mediating effect of reward-related eating, and (2) the moderating effects of anxiety, depression, and stress on the relationship between FoMO and addictive eating (Figure 1).

**Hypothesis 1.** 
*FoMO will be predictive of addictive eating.*

**Hypothesis 2.** 
*Reward-related eating will mediate the association between FoMO and addictive eating.*

**Hypothesis 3.** 
*The association between FoMO and reward-related eating, and the association between reward-related eating and addictive eating will be moderated by anxiety, depression, or perceived stress.*

Simultaneously examining these variables may provide valuable information for better understanding of the contributing factors to addictive eating and may assist in developing targeted prevention and management strategies for addictive eating behaviours.

## 2. Materials and Methods

### 2.1. Participants

Adults ≥18 years living in Australia, proficient in English, with access to the internet were invited to complete an anonymous online survey. Exclusion criteria included individuals who were pregnant or lactating. Participants were recruited, 2 July 2024 to 7 January 2025, via media releases, social media platforms, and the Join Us Research Register https://www.joinus.org.au/ (accessed 13 June 2024), a not-for-profit national research register of people in the Australian community). Additionally, participants from a previous research study examining addictive eating [43] who agreed to being recontacted, were sent an invitation to complete the survey. The study was approved by University of Newcastle Human Research Ethics Committee (H-2024-0043) and findings are reported in accordance with the Strengthening the Reporting of Observational Studies in Epidemiology (STROBE) guidelines for cross-sectional studies [44].

### 2.2. Procedure

After determining eligibility via a brief online screening questionnaire (<5 min), and online consent was obtained, eligible participants completed a 20-min survey administered in REDCap17.0.5 [45,46]. The survey captured demographics, eating behaviours (addictive eating and reward-related eating), level of FoMO, and mental health symptomology (anxiety, depression, stress). Three attention check questions were included in the survey to screen for inattentive respondents or fraudulent responses. On survey completion participants were invited to enter a prize draw to win one of two shopping vouchers (AUD 50 value each).

### 2.3. Measures

#### 2.3.1. Demographic and Anthropometric

Self-reported demographic and anthropometric information was collected via seven fixed and four open response questions. Fixed response questions included gender, years of education, employment status, marital status, and current living situation. Two further questions assessed the presence of existing health conditions and/or current use of medication which affects dietary intake or weight status, and the presence of clinically diagnosed ADHD (attention deficit hyperactivity disorder). Open response questions included age, postcode, height, and weight. Postcode was used to determine an Index of Relative Socio-Economic Disadvantage (IRSD) score, which reflects a proxy index of socioeconomic status within Australia [47]. IRSD scores range from 1 to 10 with smaller numbers reflecting areas of high disadvantage and higher numbers reflecting low disadvantage. Body Mass Index (BMI; kg/m^2^) was calculated from self-reported height and weight using standardised techniques and categorised according to the World Health Organization classifications [48].

#### 2.3.2. Addictive Eating

Addictive eating was measured using the Yale Food Addiction Scale 2.0 (YFAS 2.0) [3]. The YFAS 2.0 is a validated self-reported 35-item questionnaire that asks participants, in 8-point Likert-type format, to think of specific foods they have had difficulty controlling the consumption of within the past year. The tool assesses the presence of 11 symptoms of addictive eating with two items assessing clinically significant impairment or distress from eating. The YFAS 2.0 provides two scoring options, a ‘symptom count score’ ranging from 0 to 11, and a dichotomous FA ‘diagnosis’, when ≥2 symptoms and the clinically significant impairment or distress criteria is endorsed. Severity of the ‘diagnosis’ is classified as ‘mild’ (2–3 symptoms), ‘moderate’ (4–5 symptoms) or ‘severe’ (≥6 symptoms). The Kuder–Richardson test of internal reliability for the dichotomous scores of the 11 food addiction symptoms [49] in the present study was 0.91.

#### 2.3.3. Reward-Related Eating

Reward-related eating was measured using the Reward-Based Eating Drive Scale (RED-13) [34]. The RED-13 is a validated self-reported 13-item questionnaire, in 5-point Likert scale format from 1 (strongly disagree) to 5 (strongly agree), that assesses preoccupation with food, lack of control over eating, and lack of satiety with one factor scoring. Total scores range from 0 to 52, and higher scores reflect higher reward-based eating drive. Cronbach’s α for the sample was 0.93.

#### 2.3.4. Fear of Missing out (FoMO)

FoMO was measured using the Fear of Missing Out Scale (FoMOs) [15]. The FoMOs is a validated self-reported 10-item questionnaire, using a 5-point Likert scale from 1 (not at all true of me) to 5 (extremely true of me), that assesses the extent people are afraid they are missing out on rewarding experiences. Example questions for this scale includes ‘I get worried when I find out my friends are having fun without me’ and ‘I get anxious when I don’t know what my friends are up to’. Scores of items are summed, ranging from 10 to 50, and higher scores indicate a higher level of fear of missing out. Cronbach’s α for the sample was 0.92.

#### 2.3.5. Mental Health Status

Anxiety, depression, and stress were measured using the Generalised Anxiety Disorder scale (GAD-7) [50], the Patient Health Questionnaire (PHQ-8) [51], and the Perceived Stress Scale (PSS-4) [52], respectively. The GAD-7 is a validated self-reported seven-item questionnaire that asks the individual to rate the severity of symptoms over the past 2 weeks from 0 (not at all sure) to 3 (nearly every day). GAD-7 total scores range from 0 to 21, and severity is determined using the following cut-offs: 0–5 = mild, 6–10 = moderate, 11–15 = moderately severe, and 16–21 = severe. The PHQ-8 is a validated self-report 8-item questionnaire that asks the individual to rate the severity of depressive symptoms over the past 2 weeks from 0 (not at all) to 3 (nearly every day). Total scores range from 0 to 24, and severity is determined using the following cut-offs: 0–4 = minimal, 5–9 = mild, 10–14 = moderate, 15–19 = moderately severe, and 20–24 = severe. The PSS-4 is a validated self-report 4-item questionnaire, using a 5-point Likert scale from 1 (never) to 5 (very often), that assesses the degree to which a person perceives life as stressful. Total scores range from 0 to 16, and higher scores indicate greater stress. Currently, there is no established cut-off for the PSS-4 score to indicate adverse levels of stress. The internal reliability of the scales in the present study were: Anxiety = 0.93, Depression = 0.91, Stress = 0.74.

### 2.4. Statistical Analysis

Data analysis was performed using IBM SPSS (version 30.0). List-wise deletion was used to remove participants that had an incomplete YFAS questionnaire (>50%) or were missing an entire survey measure. Imputation methods were not used as this can introduce bias when large amounts of data are missing in important variables [53]. It was then determined that of the remaining data there were no missing data. The Kuder–Richardson test or Cronbach’s alpha was used to assess the internal consistency of the survey measures with α = 0.70 considered an acceptable lower bound. Normality of continuous variables was assessed using the Shapiro Wilk test, and visual inspections of histograms and Q–Q plots. Descriptive statistics were generated to describe the total sample and the between group differences for gender (male vs. female vs. non-binary). Continuous variables are reported as mean ±  standard deviation (SD), and categorical variables were reported as frequencies and percentages. Correlational and partial correlation analyses (Pearson correlation coefficients) were conducted as an initial evaluation of the relationships among the variables of interest.

Mediation and moderated mediation effects were assessed using the PROCESS macro for SPSS (version 4.3) developed by Hayes [54]. Prior to analyses the assumptions of multiple regression were tested. Visual inspection of plots, tolerance values greater than 0.10, and variance inflation factor values below 5 [55] for independent variables (RED-13, FoMO, GAD-7, PHQ-8, PSS scores, BMI and age) indicated that assumptions were met. Further, standardised residuals fell within the generally accepted ±3 range [56], suggesting no substantial outliers. The primary relationship of interest was between FoMO (FoMOs) and addictive eating (YFAS symptom count score). The mediator of interest was reward-related eating (RED-13 score), and the moderators of interest were (1) anxiety (GAD-7 score), (2) depression (PHQ-8 score), and (3) perceived stress (PSS-4 score). For the mediation analysis, using PROCESS Model 4, FoMO was entered as the explanatory variable (X), addictive eating entered as the outcome variable (Y), reward-related eating as the mediator (M).

For the moderated mediation analysis, using PROCESS Model 59, the model structure was replicated with anxiety or depression or stress included as moderating variable on Path a between FoMO and addictive eating, Path b between reward-related eating and addictive eating, and Path c between FoMO and addictive eating. Continuous variables were mean-centered prior to analyses. BMI was included as a covariate in all tested models to adjust for potential confounding as previous research [57] has demonstrated positive associations between BMI and addictive eating. Additional exploratory model testing was conducted with gender (categorical variable: male, female, and non-binary; noting that there is likely a lack of statistical power) and age (continuous variable) included as a covariate to obtain an unbiased estimate of the effect.

Mediation and moderated mediation effects were assessed using bias-corrected bootstrap resampled (5000) 95% confidence intervals (CI), which were considered statistically significant if the CIs did not cross zero [58]. An α < 0.05 was considered statistically significant for all other analyses. Moderation slopes were generated [59] for significant findings to graphically depict relationships between the independent variable (FoMO), mediator variable (reward-related eating), and dependent variable (addictive eating) when the levels of the moderator variable (anxiety, depression or stress) were one standard deviation below and one standard deviation above mean value of the moderator variable.

## 3. Results

### 3.1. Participant Characteristics

A total of 302 participants were recruited with 296 deemed eligible. Of these, data for 227 participants (79.3% women) are included in the current analysis. The mean age of participants was 49.4 ± 16.8 (range 18 to 88) years old. Demographic characteristics of participants are shown in Table 1. The mean BMI of participants was 28.5 ± 8.1 (range 13.8 to 66.7 kg/m^2^), with 5.3% categorised as underweight, 35.2% healthy weight, and 59.5% as being overweight or having obesity. One hundred and seventy-six participants (77.5%) had no or minimal addictive eating (defined as a YFAS symptom score of ≤1), with the remaining participants categorised as having a mild (1.8%; 2–3 symptoms), moderate (4.8%; 4–5 symptoms), or severe (15.9%; ≥6 symptoms) level of addictive eating; 50.7% of participants scored above the recommended cut-off scores for mild anxiety and 34.0% above the cut-off scores for mild depression (i.e., categorised as having moderate, moderately severe, or severe level of anxiety or depression).

### 3.2. Correlation Analysis

Descriptive statistics and correlation matrix between the measurement variables are reported in Table 2. The proposed path variables were positively correlated with one another (r = 0.52 to 0.72, all *p* < 0.001). With respect to the moderators of interest, all were (i.e., anxiety, depression, and stress) correlated positively with FoMO, reward-related eating and addictive eating (r = 0.43 to 0.62, all *p* < 0.001). BMI was associated with all variables (r = 0.15 to 0.41) with the exception of perceived stress (r = 0.13, *p* = 0.53). In addition to BMI, age was positively correlated with FoMO, anxiety, and depression (r = 0.15 to 0.29). The positive correlation between age and FoMO remained significant whilst controlling for anxiety (r = 0.23, *p* < 0.001), depression (r = 0.25, *p* < 0.001), and stress (r = 0.26, *p* < 0.001).

### 3.3. Gender Differences

Participants self-reporting as non-binary were younger, had higher mean RED-13, FoMOs, GAD-7, PHQ-8, and PSS-4 scores, and a lower mean BMI compared to men and women (Table 3). Men had lower mean YFAS symptom scores than women and non-binary participants. Mean BMI was similar across men and women.

### 3.4. Mediation Analysis

**Figure 2:**
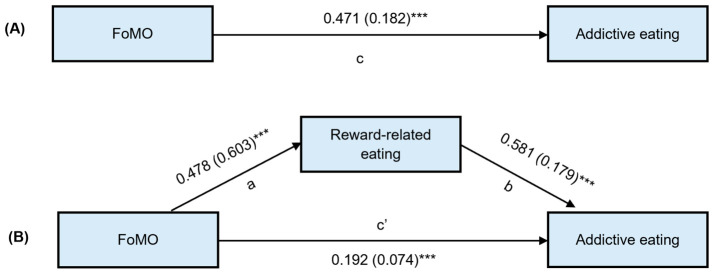
The mediating effect of reward-related eating in the relationship between FoMO and addictive eating, accounting for BMI. (**A**) The total effect (path c). (**B**) The effect of FoMO on reward-related eating (path a); the effect of reward-related eating on addictive eating (path b); and the direct effect of FoMO on addictive eating, adjusted by the mediator (path c′). Standardised regression coefficients are reported with unstandardised coefficients in brackets. *** *p* < 0.001.

The mediation analysis revealed the total effect of the model (R^2^ = 0.349, F_(3,223)_ = 60.043, *p* < 0.001) was significant supporting hypothesis 1 that FoMO is a significant predictor of addictive eating (path c: *B* = 0.182, SE = 0.021, t = 8.628, *p* < 0.001, LLCI 0.141, ULCI 0.224), Figure 2A. The direct effect of FoMO on addictive eating, when the mediating variable was included (R^2^ = 0.555, F_(3,223)_ = 92.631, *p* < 0.001), reduced the effect but remained significant (path c′: *B* = 0.074, SE = 0.020, t = 3.637, *p* < 0.001, LLCI 0.034, ULCI 0.115), Figure 2B. The indirect effects, with examination of the bias-corrected bootstrap 95% confidence intervals, were also significant (β = 0.108, SE = 0.017, LLCI 0.077, ULCI 0.142), with FoMO having a predictive effect on reward-related eating (path a: *B* = 0.603, SE = 0.066, t = 9.076, *p* < 0.001, LLCI 0.472, ULCI 0.734), and reward-related eating having a predictive effect on addictive eating (path b: *B* = 0.179, SE = 0.018, *p* < 0.001, LLCI 0.144, ULCI 0.213). When gender or age were included as a covariate in the model, these findings remained significant. These results suggest that reward-related eating partially mediates the relationship between FoMO and addictive eating (Hypothesis 2).

### 3.5. Moderated Mediation Analysis

**Figure 3:**
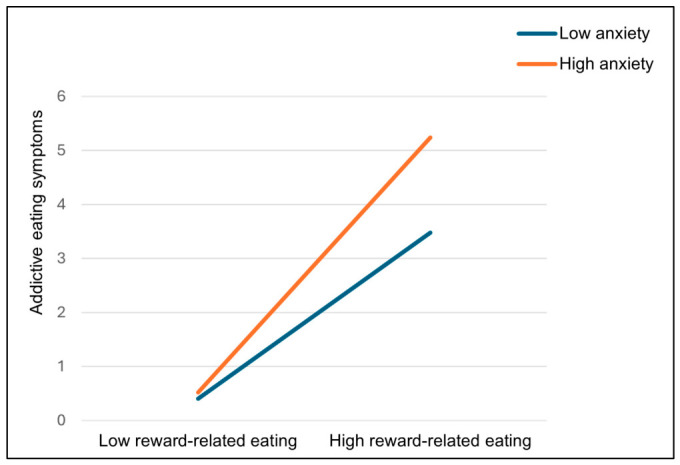
Moderation of the effect of reward-related eating on addictive eating at low (−1 SD) and high (+1 SD) values of the moderator anxiety.

The moderated mediation analyses (Table 4), controlling for BMI (R^2^ = 0.457, F_(4,222)_ = 46.803, *p* < 0.001), revealed that anxiety had a significant moderating effect on the relationship between reward-related eating and addictive eating (Path b: *B* = 0.007, SE = 0.003, t = 2.078, *p* = 0.039, LLCI 0.000, ULCI 0.013). The conditional effect was the strongest for high values (+1 SD) of anxiety (*B* = 0.211, SE = 0.028, t = 7.445, *p* < 0.001, LLCI 0.155, ULCI 0.267), it was weaker but still significant for medium values (mean) and for small values (−1 SD) of anxiety (*B* = 0.138, SE = 0.022, t = 6.210, *p* < 0.001, LLCI 0.094, ULCI 0.181), Figure 3. The proportion of variance explained by the interaction effect was modest (ΔR^2^ = 0.008, F_(1,220)_ = 4.317, *p* = 0.039).

When gender or age were also included as a covariate in the model (see Appendix A, Table A1 and Table A2), this finding remained significant (Path b: *B* = 0.006, SE = 0.003, t = 2.005, *p* = 0.046, LLCI 0.000, ULCI 0.012; and B = 0.006, SE = 0.003, t = 2.053, *p* = 0.041, LLCI 0.000, ULCI 0.013, respectively). The proportion of variance explained by the interaction effect in each of the models was modest (ΔR^2^ = 0.007, F_(1,219)_ = 4.018, *p* = 0.046; and ΔR^2^ = 0.008, F_(1,219)_ = 4.215, *p* = 0.041, respectively). These results provide some support for hypothesis 3, but there was no evidence for anxiety as a moderator between FoMO and reward-related eating (path a) or between FoMO and addictive eating (path c′). Depression and stress did not significantly moderate any of the relationships (path a, b or c′), Table 4.

## 4. Discussion

This study explored the potential role of FoMO in addictive eating. The findings indicate there is evidence of a direct relationship between FoMO and addictive eating. Further, across genders, greater levels of FoMO influences the risk of reward-related eating, which in turn is associated with higher addictive eating symptoms. These preliminary findings suggest that anxiety severity may be an important risk factor linking increased reward-related eating to increased addictive eating symptoms. While statistically significant, the moderating effect of anxiety was small. This modest value was expected due to the multifactorial nature of disordered eating outcomes which are influenced by genetic, environmental, and behavioural factors. The results indicate other contributors may play a stronger part in the relationship between reward-related eating and addictive eating. However, given that the current study, and past research [6,11,13,60], has found associations exist between reward-related eating, addictive eating, and anxiety scores, the clinical relevance of the observed effect should not be overlooked. In the current sample there was a lack of evidence for a moderating effect of depression and stress. Potentially the PSS-4 and PHQ-8 may have lower sensitivity to detect moderation effects. It should also be noted that the study was exploratory and therefore may not be statistically powered to detect effects. Future research in larger samples, with an a priori sample size calculation, with moderators tested simultaneously in one model, is needed to explore these possibilities further. The present study is the first to explore FoMO in relation to reward-related eating and addictive eating. While the findings are preliminary, they may be useful for future research and conceptualising more targeted treatments to reduce addictive eating in vulnerable individuals.

There are several reasons FoMO could be linked to food and addictive eating including social eating, peer influence, and engagement with social media. Individuals may experience heightened FoMO in social settings, especially when food is involved. At events, individuals may eat more or choose foods they would not normally eat in a desire to fit in and avoid exclusion. The constant exposure to the enormous amount of food-related content online may drive food envy and trigger FoMO. For example, seeing others post about indulgent meals or trending restaurants on social media platforms can trigger a desire to join in. Further, seeing others enjoying food experiences may lead to cravings or a sense of deprivation and result in mimicking behaviours, like ordering the same food or trying viral recipes.

The influence of FoMO is increasingly being utilised by marketers to promote food products, with tactics such as scarcity and limited-time-offers commonly used to create a sense of urgency [61]. When a food product is only available in limited quantities or for a short period, it triggers FoMO and can make even the most mundane food seem more desirable. The anticipation of missing out can drive people to seek immediate gratification through the consumption of food which can lead to overeating. This overlaps with behavioural mechanisms seen in addictive eating research [62]. The current study provides support for the theory that some individuals may consume food because they fear missing the opportunity. Future research could consider how these concepts or behavioural approaches may contribute to the management of a path towards personalised smart nutrition.

Emotional eating has long been associated with increased food intake [63], and individuals with addictive eating have previously expressed that they often eat or consume food for reasons other than hunger [8,14]. For example, eating in response to emotional states, such as feeling anxious or feeling stressed [14]. FoMO may heighten anxiety or stress, and for some eating may become a way to soothe the discomfort of feeling left out or disconnected engendered by FoMO. These maladaptive coping mechanisms could lead to impulsive or binge-eating behaviours.

Past research has found that individuals with anxiety disorders exhibit a higher likelihood of experiencing FoMO compared to those without anxiety disorders [38,64]. Similarly, in the current study, of the mental health states researched, anxiety showed the most consistent relationship. While not measured in the current study, it has been hypothesised that anxiety is closely linked to FoMO with both involving anticipatory emotions and social comparison, which can activate similar psychological and neurological pathways in brain regions [17,37]. There may also be neurobiological overlap with both conditions involving the pre-frontal cortex, an area of the brain that regulates emotions, decision-making, and behaviour [27,65]. Further, both FoMO and anxiety activate the amygdala and dopaminergic systems, brain regions that are involved in threat detection and reward anticipation [17,65,66]. This overlap could make FoMO feel like a low-grade threat, triggering anxious responses. This may be further exacerbated in vulnerable individuals, such as those with neurodevelopmental conditions who may experience atypical dopaminergic signaling [67].

While the current study found FoMO scores to be similar across men and women, previous research suggests that gender differences may exist, though these differences appear to be context dependent. For example, women may be more sensitive to social exclusion attributed to food with previous studies linking FoMO in women to lower perceived social support [68]. Whereas men may experience FoMO in ways more closely associated with competitive or status-driven contexts [69,70], such as gaming or achievement-oriented activities [24,68]. These nuanced patterns indicate that the effect of gender warrants further exploration in future studies to determine whether the relationship between FoMO, reward-related eating, and addictive eating is moderated by gender.

In the current study, age was positively correlated with FoMO. This differs from many previous studies [15,25,71,72] with higher levels of FoMO reported among younger individuals with levels decreasing as age increases. However, research examining FoMO in adults over the age of 45 years remains limited [73], leaving a gap in understanding how FoMO manifests later in life. While age was also positively correlated with anxiety and depression in the current study, mental health status (anxiety, depression and stress) had little influence in controlling for the relationship between FoMO and age. However, it is theoretically plausible that the underlying drivers of FoMO may shift over time. For example, missing out on food-related activities could become more important for older adults who report higher levels of mental health symptomology, potentially reflecting a greater reliance on social and communal experiences for emotional wellbeing. The speculative hypothesis that age-related changes in social priorities and mental health may influence the nature of FoMO, warrants further investigation.

The following limitations need to be taken into account when interpreting the findings. The sample was from Australia, predominantly female, and only a small number of individuals reported moderate to severe addictive eating. Therefore, results may not be may not be generalisable to the broader population, to other ethnicities, or to those with extreme addictive eating behaviours. Insignificant findings could be attributed to the modest sample size and the adequacy of statistical power to detect small interaction effects. Additionally, the unequal balance in groups for gender, which may produce underpowered study results and limits the ability to conduct inferential statistical testing to explore gender differences. Using a larger sample size may overcome this potential limitation. Therefore, it would be useful for future research to replicate the study with larger more representative samples. Furthermore, self-report measures were used to collect data, and current FoMO scales are context-bound to social media and not specifically related to food. Integration with qualitative techniques in a multi-method approach is recommended for future research to help overcome this issue. While the current study was exploratory and cross-sectional, the different time scales used by the measures to assess symptomology of outcome and moderator variables should be considered in future studies and adjustments made to the reporting timeperiod, particularly if using these tools to evaluate change over time. Due to the high intercorrelations among the mental health variables, the separate coefficients should be interpreted with caution. While mental health states (anxiety, depression, stress) have shared causes and can occur simultaneously or in isolation, use of an alternative tool (e.g., Depression Anxiety Stress Scale [74]) may be better in future to provide a composite mental health score for analysis in a single model. It should also be noted that the FoMO scale used in the current study has been validated in predominantly younger samples, and therefore may not have measured the construct with equivalent validity across the wide age range. The current study relied on self-reported height and weight data to calculate BMI which has the potential for reporting bias and inaccuracy in these data may affect the precision of the estimated interaction and mediation effects. While self-reported height and weight have been shown to be a valid measure of weight status in the general population [75,76], there can be a tendency for modest misestimations in height and weight among individuals with greater BMI [76,77] and those with disordered eating patterns [78,79]). Therefore, findings should be viewed in light of this. Finally, the cross-sectional design of this study requires caution in interpreting causality. Future longitudinal studies with multiple data collection timepoints are needed to replicate findings.

From a clinical implication perspective, existing intervention strategies have demonstrated varying degrees of effectiveness in addressing FoMO and its associated symptoms. For example, cognitive-behavioural therapy (CBT) with a focus on identifying and modifying dysfunctional cognitions and behaviours [80], has shown promise in reducing FoMO severity [81,82]. These findings suggest that targeting maladaptive thought patters and enhancing coping strategies may help mitigate FoMO-related distress. However, future research is needed to better understand the relationship between FoMO and addictive eating, particularly through longitudinal designs that can clarify the directionality of these associations. Such research will be critical for informing tailored interventions that address FoMO within the context of disordered eating, potentially integrating behavioural and cognitive approaches to reduce vulnerability to reward-driven or compulsive eating behaviours.

## 5. Conclusions

The findings suggest that individuals with higher intensity of FoMO may experience greater reward-related eating and greater addictive eating. Furthermore, the moderating effect of anxiety underscores the importance of considering mental health factors in understanding the impact of reward-related eating on addictive eating outcomes. While the current study is exploratory and further research is needed, these preliminary findings may be considered in the development of interventions aimed at mitigating the negative effects of FoMO on eating behaviours, particularly among populations vulnerable to anxiety.

## Figures and Tables

**Table 1:** Characteristics of participants (n = 227).

Variable	n (%) or Mean ± SD (Range)
Gender	
Man	42 (18.5)
Woman	180 (79.3)
Other/non-binary	5 (2.2)
Health condition	
No	161 (70.9)
ADHD	
No	207 (91.2)
Aboriginal or Torres Strait Islander descent	
No	224 (98.7)
Decile (postcode) ^	6.6 ± 2.5 (1–10) *n* = 224
Marital status	
Single, never married	61 (26.9)
In a relationship, not married	28 (12.3)
Married or in a domestic partnership	104 (45.8)
Divorced or separated	24 (10.6)
Widowed	10 (4.4)
Current living situation	
Renting	59 (26.0)
Own home	133 (58.6)
Family home	23 (10.1)
Supported accommodation or department of housing	6 (2.6)
Other	6 (2.6)
Currently living with ^^	
Parent/s	23 (10.1)
Sibling/s	16 (7.0)
Partner	116 (51.1)
Children	55 (24.2)
Flatmates or friends	15 (6.6)
Alone	60 (26.4)
Other	5 (2.2)
Highest qualification	
School certificate (Year 10 or equivalent)	9 (4.0)
Higher school certificate (Year 12 or equivalent)	25 (11.0)
Trade or diploma	44 (19.4)
Undergraduate university degree	75 (33.0)
Postgraduate university degree (e.g., Master’s)	57 (25.1)
Higher research degree (e.g., PhD)	16 (7.0)
Employment status	
Employed—Full time	73 (32.2)
Employed—Part time	35 (15.4)
Employed—Casual	25 (11.0)
Student	24 (10.6)
Unemployed—looking for work	10 (4.4)
Unemployed—not looking for work	60 (26.4)
Household income	
AUD 0–30 K	33 (14.5)
AUD 31 K–60 K	44 (19.4)
AUD 61 K–90 K	22 (9.7)
AUD 91 K–120 K	30 (13.2)
AUD 120 K +	74 (32.6)
Prefer not to say	24 (10.6)

ADHD, attention deficit hyperactivity disorder. ^ Postcode not reported by n = 3 participants. ^^ Percentages exceed 100% as participants were able to select multiple responses.

**Table 2:** Descriptive statistics and correlational analysis of study variables.

		Mean	SD	Range	1	2	3	4	5	6	7	
1	YFAS 2.0	2.8	3.4	0–11	–							
2	RED-13	25.3	11.2	0–52	0.72 ***	–						
3	FoMOs	20.3	8.9	10–50	0.52 ***	0.53 ***	–					
4	GAD7	6.9	5.6	0–21	0.54 ***	0.52 ***	0.55 ***	–				
5	PHQ8	7.7	6.2	0–24	0.62 ***	0.60 ***	0.54 ***	0.81 ***	–			
6	PSS-4	7.0	3.4	0–16	0.43 ***	0.44 ***	0.46 ***	0.70 ***	0.68 ***	–		
7	BMI	28.5	8.1	13.8–66.7	0.36 ***	0.41 ***	0.15 *	0.15 *	0.20 **	0.13	–	
8	Age	49.7	16.8	18–88	0.02	0.07	0.29 **	0.18 **	0.15 *	0.13	0.20 *	–

Pearsons’s correlation coefficient. * *p* < 0.05, ** *p* < 0.01, *** *p* < 0.001. YFAS 2.0, Yale Food Addiction Scale; RED-13, Reward-Based Eating Drive Scale; FoMOs, FoMO Scale, Fear of Missing Out Scale; GAD7, Generalised Anxiety Disorder-7 item; PHQ8, Patient Health Questionnaire-8 item; PSS-4, Perceived Stress Scale-4 item; BMI, Body Mass Index.

**Table 3:** Descriptive statistics of study variables by gender.

Variable	Women (n = 150)	Men (n = 42)	Non-Binary (n = 5)
		Mean ± SD (range)	
BMI	28.6 ± 8.5 (13.8–66.7)	28.4 ± 6.5 (18.1–46.8)	26.3 ± 6.5 (18.7–33.4)
Age	49.1 ± 16.2 (18–88)	53.6 ± 17.9 (18–76)	24.8 ± 7.8 (18–35)
YFAS 2.0	3.0 ± 3.5 (0–11)	1.9 ± 3.0 (0–11)	2.6 ± 2.0 (0–5)
RED-13	25.5 ± 11.5 (0–52)	23.7 ± 9.5 (6–41)	32.0 ± 8.7 (21–44)
FoMOs	20.3 ± 8.8 (10–50)	20.1 ± 9.2 (10–43)	24.8 ± 6.9 (14–33)
GAD-7	6.7 ± 5.6 (0–21)	6.8 ± 5.8 (0–19	12.4 ± 4.5 (7–19)
PHQ-8	7.8 ± 6.3 (0–24)	6.8 ± 5.9 (0–21)	11.8 ± 3.6 (7–17)
PSS-4	6.9 ± 3.4 (0–16)	7.5 ± 3.5 (1–15)	9.0 ± 1.2 (8–11)

YFAS 2.0, Yale Food Addiction Scale; RED-13, Reward-Based Eating Drive Scale; FoMOs, FoMO Scale, Fear of Missing Out Scale; GAD-7, Generalised Anxiety Disorder-7 item; PHQ-8, Patient Health Questionnaire-8 item; PSS-4, Perceived Stress Scale-4 item; BMI, Body Mass Index.

**Table 4:** Moderated mediation effects of anxiety, depression and stress on the relationship between FoMO, reward-related eating and addictive eating (n = 227), controlling for BMI.

Moderator	Path		*B*	SE	t	*p*	95%CI
Anxiety	a	FoMO → Reward-related eating	0.445	0.080	5.547	<0.001 **	[0.287, 0.604]
		Anxiety	0.614	0.119	5.164	<0.001 **	[0.380, 0.848]
		FoMO × Anxiety	−0.017	0.011	−1.566	0.119	[−0.038, 0.004]
		BMI	0.427	0.069	6.168	<0.001 **	[0.291, 0.564]
	b & c′	Reward-related eating → Addictive eating	0.174	0.018	9.538	<0.001 **	[0.138, 0.210]
		FoMO → Addictive eating, with RRE as a mediator	0.022	0.023	0.953	0.341	[−0.023, 0.066]
		Anxiety	0.083	0.034	2.445	0.015 *	[0.016, 0.150]
		Reward-related eating × Anxiety	0.007	0.003	2.078	0.039 *	[0.000, 0.013]
		FoMO × Anxiety	0.005	0.003	1.478	0.141	[−0.002, 0.012]
		BMI	0.042	0.020	2.084	0.038 *	[0.002, 0.081]
Depression	a	FoMO → Reward-related eating	0.379	0.075	5.050	<0.001 **	[0.231, 0.527]
		Depression	0.724	0.102	7.061	<0.001 **	[0.522, 0.926]
		FoMO × Depression	−0.012	0.010	−1.302	0.194	[−0.031, 0.006]
		BMI	0.388	0.067	5.798	<0.001 **	[0.256, 0.519]
	b & c′	Reward-related eating → Addictive eating	0.152	0.019	8.139	<0.001 **	[0.115, 0.189]
		FoMO → Addictive eating, with RRE as a mediator	0.027	0.022	1.232	0.219	[0-.016, 0.070]
		Depression	0.120	0.033	3.695	<0.001 **	[0.056, 0.185]
		Reward-related eating × Depression	0.004	0.003	1.327	0.186	[−0.002, 0.009]
		FoMO × Depression	0.005	0.003	1.699	0.091	[−0.001, 0.011]
		BMI	0.043	0.020	2.165	0.031 *	[0.004, 0.082]
Stress	a	FoMO → Reward-related eating	0.513	0.078	6.603	<0.001 **	[0.360, 0.666]
		Stress	0.724	0.189	3.824	<0.001 **	[0.351, 1.098]
		FoMO × Stress	−0.024	0.021	−1.189	0.236	[−0.065, 0.016]
		BMI	0.446	0.071	6.322	<0.001 **	[0.307, 0.586]
	b & c′	Reward-related eating → Addictive eating	0.177	0.018	9.930	<0.001 **	[0.142, 0.212]
		FoMO → Addictive eating, with RRE as a mediator	0.036	0.022	1.595	0.112	[−0.008, 0.080]
		Stress	0.116	0.052	2.250	0.025 *	[0.014, 0.218]
		Reward-related eating x Stress	0.007	0.005	1.441	0.151	[−0.003, 0.017]
		FoMO × Stress	0.012	0.007	1.850	0.066	[−0.001, 0.025]
		BMI	0.037	0.020	1.835	0.068	[−0.003, 0.077]

RRE, reward-related eating. *B* = unstandardised regression coefficients; SE = standard error; CI = confidence interval. * *p* < 0.05, ** *p* < 0.001. Moderators—Anxiety: model for a-path R^2^ = 0.457, F_(4,222)_ = 46.803, *p* < 0.001, model for b-path and c′-path R^2^ = 0.603, F_(6,220)_ = 55.587, *p* < 0.001. Depression: model for a-path R^2^ = 0.503, F_(4,222)_ = 56.272, *p* < 0.001. Model for b-path and c′-path R^2^ = 0.610, F_(6,220)_ = 57.302, *p* < 0.001. Stress: model for a-path R^2^ = 0.433, F_(4,222)_ = 42.460, *p* < 0.001, model for b-path and c′-path R^2^ = 0.586, F_(6,220)_ = 51.936, *p* < 0.001.

## Data Availability

Data are available from the corresponding author on reasonable request due to privacy and ethical restrictions.

## Abbreviations

The following abbreviations are used in this manuscript:

FoMO	Fear of Missing Out
ADHD	Attention Deficit Hyperactivity Disorder
IRSD	Index of Relative Socio-Economic Disadvantage
BMI	Body Mass Index
YFAS	Yale Food Addiction Scale
RED-13	Reward-Based Eating Drive Scale-13 item
FoMOs	Fear of Missing Out Scale
GAD-7	Generalised Anxiety Disorder-7 item
PHQ-8	Patient Health Questionnaire-8 item
PSS-4	Perceived Stress Scale-4 item

## Appendix A

**Table A1:** Moderated mediation effects of anxiety, depression, and stress on the relationship between FoMO, reward-related eating, and addictive eating (n = 227), controlling for BMI and gender.

Moderator	Path		*B*	SE	t	*p*	95%CI
Anxiety	a	FoMO → Reward-related eating	0.440	0.080	5.484	<0.001 **	[0.282, 0.599]
		Anxiety	0.605	0.119	5.087	<0.001 **	[0.371, 0.840]
		FoMO × Anxiety	−0.015	0.011	−1.415	0.159	[−0.036, 0.006]
		BMI	0.429	0.069	6.196	<0.001 **	[0.293, 0.566]
		Gender	1.504	1.308	1.150	0.251	[−1.074, 4.082]
	b & c′	Reward-related eating → Addictive eating	0.172	0.018	9.400	<0.001 **	[0.136, 0.208]
		FoMO → Addictive eating, with RRE as a mediator	0.021	0.023	0.923	0.357	[−0.024, 0.065]
		Anxiety	0.082	0.034	2.419	0.016 *	[0.015, 0.149]
		Reward-related eating × Anxiety	0.006	0.003	2.005	0.046 *	[0.000, 0.012]
		FoMO × Anxiety	0.006	0.003	1.638	0.103	[−0.001, 0.012]
		BMI	0.043	0.020	2.161	0.032 *	[0.004, 0.083]
		Gender	0.468	0.347	1.349	0.179	[−0.216, 1.152]
Depression	a	FoMO → Reward-related eating	0.378	0.075	5.021	<0.001 **	[−0.229, 0.526]
		Depression	0.715	0.103	6.934	<0.001 **	[0.512, 0.918]
		FoMO × Depression	−0.011	0.010	−1.189	0.236	[−0.030, 0.008]
		BMI	0.389	0.067	5.818	<0.001 **	[0.259, 0.521]
		Gender	1.025	1.258	0.815	0.416	[−1.454, 3.504]
	b & c′	Reward-related eating → Addictive eating	0.150	0.019	8.043	<0.001 **	[0.114, 0.187]
		FoMO → Addictive eating, with RRE as a mediator	0.026	0.022	1.208	0.228	[−0.017, 0.070]
		Depression	0.119	0.033	3.653	<0.001 **	[0.055, 0.183]
		Reward-related eating × Depression	0.003	0.003	1.210	0.228	[−0.002, 0.009]
		FoMO × Depression	0.006	0.003	1.850	0.066	[0.000, 0.012]
		BMI	0.044	0.020	2.235	0.026 *	[0.005, 0.084]
		Gender	0.382	0.346	1.104	0.271	[−0.300, 1.065]
Stress	a	FoMO → Reward-related eating	0.504	0.077	6.499	<0.001 **	[0.351, 0.656]
		Stress	0.742	0.189	3.929	<0.001 **	[0.370, 1.115]
		FoMO × Stress	−0.023	0.020	−1.131	0.259	[−0.063, 0.017]
		BMI	0.447	0.070	6.359	<0.001 **	[0.309, 0.586]
		Gender	2.251	1.323	1.701	0.090	[−0.358, 4.859]
	b & c′	Reward-related eating → Addictive eating	0.174	0.018	9.709	<0.001 **	[0.139, 0.209]
		FoMO → Addictive eating, with RRE as a mediator	0.035	0.022	1.572	0.117	[−0.009, 0.079]
		Stress	0.121	0.052	2.339	0.020 *	[0.019, 0.223]
		Reward-related eating × Stress	0.006	0.005	1.286	0.200	[−0.003, 0.016]
		FoMO × Stress	0.013	0.007	1.956	0.052	[0.000, 0.026]
		BMI	0.039	0.020	1.903	0.058	[−0.001, 0.079]
		Gender	0.431	0.355	1.212	0.227	[−0.270, 1.131]

RRE, reward-related eating. *B* = unstandardised regression coefficients; SE = standard error; CI = confidence interval. * *p* < 0.05, ** *p* < 0.001. Moderators—Anxiety: model for a-path R^2^ = 0.461, F_(5,221)_ = 37.761, *p* < 0.001, model for b-path and c′-path R^2^ = 0.606, F_(7,219)_ = 48.083, *p* < 0.001. Depression: model for a-path R^2^ = 0.505, F_(5,221)_ = 45.082, *p* < 0.001. Model for b-path and c′-path R^2^ = 0.612, F_(7,219)_ = 49.339, *p* < 0.001. Stress: model for a-path R^2^ = 0.441, F_(5,221)_ = 34.836, *p* < 0.001, model for b-path and c′-path R^2^ = 0.589, F_(7,219)_ = 44.821, *p* < 0.001.

**Table A2:** Moderated mediation effects of anxiety, depression, and stress on the relationship between FoMO, reward-related eating, and addictive eating (n = 227), controlling for BMI and age.

Moderator	Path		*B*	SE	t	*p*	95%CI
Anxiety	a	FoMO → Reward-related eating	0.458	0.084	5.437	<0.001 **	[0.292, 0.624]
		Anxiety	0.618	0.119	5.178	<0.001 **	[0.383, 0.854]
		FoMO × Anxiety	−0.018	0.011	−1.625	0.106	[−0.039, 0.004]
		BMI	0.417	0.072	5.777	<0.001 **	[0.275, 0.560]
		Age	0.018	0.036	0.501	0.617	[−0.053, 0.089]
	b & c′	Reward-related eating → Addictive eating	0.174	0.018	9.493	<0.001 **	[0.138, 0.210]
		FoMO → Addictive eating, with RRE as a mediator	0.029	0.024	1.214	0.226	[−0.018, 0.075]
		Anxiety	0.086	0.034	2.525	0.012	[0.019, 0.153]
		Reward-related eating × Anxiety	0.006	0.003	2.053	0.041 *	[0.000, 0.013]
		FoMO × Anxiety	0.005	0.003	1.332	0.184	[−0.002, 0.011]
		BMI	0.036	0.021	1.770	0.078	[−0.004, 0.077]
		Age	0.010	0.010	1.043	0.298	[−0.009, 0.029]
Depression	a	FoMO → Reward-related eating	0.389	0.079	4.933	<0.001 **	[0.234, 0.545]
		Depression	0.726	0.1036	7.060	<0.001 **	[0.523, 0.928]
		FoMO × Depression	−0.013	0.010	−1.348	0.179	[−0.032, 0.006]
		BMI	0.379	0.070	5.446	<0.001 **	[0.242, 0.517]
		Age	0.015	0.035	0.422	0.674	[−0.053, 0.083]
	b & c′	Reward-related eating → Addictive eating	0.151	0.019	8.101	<0.001 **	[0.114, 0.188]
		FoMO → Addictive eating, with RRE as a mediator	0.035	0.023	1.512	0.132	[−0.010, 0.080]
		Depression	0.123	0.033	1.562	0.120	[0.058, 0.187]
		Reward-related eating × Depression	0.003	0.003	1.285	0.200	[−0.002, 0.009]
		FoMO x Depression	0.005	0.003	1.562	0.120	[−0.001, 0.011]
		BMI	0.037	0.020	1.825	0.069	[−0.003, 0.078]
		Age	0.011	0.009	1.147	0.253	[−0.008, 0.029
Stress	a	FoMO → Reward-related eating	0.514	0.081	6.332	<0.001 **	[0.354, 0.674]
		Stress	0.725	0.190	3.816	<0.001 **	[0.350, 1.099]
		FoMO × Stress	−0.024	0.021	−1.189	0.236	[−0.065, 0.016]
		BMI	0.445	0.073	6.072	<0.001 **	[0.301, 0.589]
		Age	0.003	0.036	0.076	0.940	[−0.069, 0.075]
	b & c′	Reward-related eating → Addictive eating	0.177	0.018	9.928	<0.001 **	[0.142, 0.212]
		FoMO → Addictive eating, with RRE as a mediator	0.043	0.023	1.859	0.064	[−0.003, 0.089]
		Stress	0.117	0.052	2.262	0.025 *	[0.015, 0.219]
		Reward-related eating × Stress	0.007	0.005	1.383	0.168	[−0.003, 0.016]
		FoMO × Stress	0.012	0.007	1.839	0.067	[−0.001, 0.025]
		BMI	0.031	0.021	1.501	0.135	[−0.010, 0.072]
		Age	0.012	0.010	1.205	0.229	[−0.007, 0.031]

RRE, reward-related eating. *B* = unstandardised regression coefficients; SE = standard error; CI = confidence interval. * *p* < 0.05, ** *p* < 0.001. Moderators—Anxiety: model for a-path R^2^ = 0.458, F_(5,221)_ = 37.66, *p* < 0.001, model for b-path and c′-path R^2^ = 0.605, F_(7,219)_ = 47.820, *p* < 0.001. Depression: model for a-path R^2^ = 0.504, F_(5,221)_ = 44.886, *p* < 0.001. Model for b-path and c′-path R^2^ = 0.612, F_(7,219)_ = 49.374, *p* < 0.001. Stress: model for a-path R^2^ = 0.433, F_(5,221)_ = 33.817, *p* < 0.001, model for b-path and c′-path R^2^ = 0.589, F_(7,219)_ = 44.815, *p* < 0.001.
